# Femtosecond Two-Photon Absorption Spectroscopy of Poly(fluorene) Derivatives Containing Benzoselenadiazole and Benzothiadiazole

**DOI:** 10.3390/ma10050512

**Published:** 2017-05-07

**Authors:** Marcelo Gonçalves Vivas, Ruben Dario Fonseca, Jonathas de Paula Siqueira, Cleber Renato Mendonça, Paula C. Rodrigues, Leonardo De Boni

**Affiliations:** 1 Instituto de Ciência e Tecnologia, Universidade Federal de Alfenas, Poços de Caldas, MG 37715-400, Brazil; mavivas82@gmail.com; 2 Instituto de Física de São Carlos, Universidade de São Paulo, São Carlos, SP 13566-590, Brazil; rubenzfonseca@gmail.com (R.D.F.); jonathasusp@gmail.com (J.D.P.S.); deboni@ifsc.usp.br (L.D.B.); 3 Departamento de Ciencias Naturales y Exactas, Universidad de la Costa, Barranquilla 080002, Colombia; 4 Departamento Acadêmico de Química e Biologia, Universidade Tecnológica Federal do Paraná, Curitiba, PR 81280-340, Brazil; paulac@utfpr.edu.br

**Keywords:** two-photon absorption, organic materials, polymers, tunable Z-Scan technique, nonlinear optics

## Abstract

We have investigated the molecular structure and two-photon absorption (2PA) properties relationship of two push–pull poly(fluorene) derivatives containing benzoselenadiazole and benzothiadiazole units. For that, we have used the femtosecond wavelength-tunable Z-scan technique with a low repetition rate (1 kHz) and an energy per pulse on the order of nJ. Our results show that both 2PA spectra present a strong 2PA (around 600 GM (1 GM = 1 × 10^−50^ cm^4^·s·photon^−1^)) band at around 720 nm (transition energy 3.45 eV) ascribed to the strongly 2PA-allowed 1Ag-like → mAg-like transition, characteristic of poly(fluorene) derivatives. Another 2PA band related to the intramolecular charge transfer was also observed at around 900 nm (transition energy 2.75 eV). In both 2PA bands, we found higher 2PA cross-section values for the poly(fluorene) containing benzothiadiazole unit. This outcome was explained through the higher charge redistribution at the excited state caused by the benzothiadiazole group as compared to the benzoselenadiazole and confirmed by means of solvatochromic Stokes shift measurements. To shed more light on these results, we employed the sum-over-states approach within the two-energy level model to estimate the maximum permanent dipole moment change related to the intramolecular charge transfer transition.

## 1. Introduction

Semiconductor polymers as poly(fluorene) derivatives have been studied in recent years for their interesting molecular design for the development of novel electronic and optical devices [1]. Basically, semiconductor polymers present three important features that prompt them as remarkable candidates for fabricating polymeric photonic devices [2,3]: (i) their high molar absorptivity [1], (ii) their high photoluminescence and electroluminescence quantum yield [4,5], and (iii) the excellence of their materials for thin film preparation [6]. At the same time, semiconductor polymers are fascinating materials for studying nonlinear optical effects such as two-photon absorption (2PA). Organic materials for 2PA are interesting for applications in 3D micro-fabrication and micromachining [7,8,9,10], 3D data storage [11,12], optical power limiting [13,14], two-photon induced photopolymerization [15], two-photon pumped laser [16], and so on. The key feature for such applications is the quadratic dependence of the 2PA process on the excitation irradiance, which provides spatial confinement of excitation [17]. Furthermore, the 2PA process occurs, in general, at the near infrared region in which the light scattering strength is smaller than that in the ultraviolet and visible regions (linear absorption regime).

As a matter of fact, 2PA cross sections in semiconductor polymers are closely related to the effective numbers of π-electrons [18], the π-conjugation length [19], and the charge separation induced by introducing electron-donating and withdrawing groups [20]. These effects are associated with the transition dipole moment (μ) and dipole moment change (Δμ), which govern the 2PA cross section in organic materials [21]. However, although increasing the polymeric chain seems like an interesting option to increase the π-conjugation length, which in turn would increase the nonlinearity, after a given point the polymeric structure start to bend, strongly affecting the π-conjugation planarization, causing a great reduction in the 2PA cross section [22]. In this context, push–pull polymers have gained attention because their structure allows for the obtainment of great charge redistribution at the excited state, providing a prodigious nonlinear optical response. This behavior is possibly due to the ability of synthesizing copolymers having a delocalized π-system consisting of alternating electron-rich (donor) and electron-deficient (acceptor) repeating units. Therefore, the difference in electronic density between donor and acceptor units allows the band gap to be narrowed or widened, based on the choice of the units. A fine tuning of the optical and electronic properties of polymers containing thiophene and its derivatives can be made through the exchange of the sulfur atom by another chalcogen atom (selenium or tellurium) [23,24]. Therefore, the characterization of the nonlinear optical response of organic materials containing different donor–electron and acceptor groups is very important for obtaining information on promising molecular designs that can be used to improve the nonlinear optical response and decrease the irradiance threshold necessary to develop novel photonics devices based on two-photon absorption, for instance.

In this work, we have studied the femtosecond 2PA spectra of two donor-π-acceptor poly(fluorene) derivative polymers, whose monomer molecular structures can be seen in Section 3, by using the wavelength-tunable Z-scan technique with a low energy per pulse (nJ) and repetition rate (1 kHz). The first sample is the poly[9,9-bis(3’-(tert-butyl propanoate))fluorene-co-4,7-(2,1,3-benzoselenadiazole)] (PFeBSe), and the second one is the poly[9,9-bis(3’-(tert-butyl propanoate))fluorene-co-4,7-(2,1,3-benzothiadiazole)] (PFeBT). The difference between these polymers is the change from the benzothiadiazole group to the benzoselenadiazole group, because, in general, the substitution of Se for S tends to increase the push–pull strength of the molecular structure. To the best of our knowledge, 2PA on these two polymers has not yet been published. All 2PA bands observed along the nonlinear spectra were described based on similar results published in the literature for polyfluorene [16,17,19]. In addition to the analyses of the 2PA effect, the maximum permanent dipole moment change related to the intramolecular charge transfer transition was estimated through the sum-over-states approach within the two-energy level model. Solvatochromic measurements were also performed and helped us better understand the 2PA results. 

## 2. Results and Discussion

**Figure 1:**
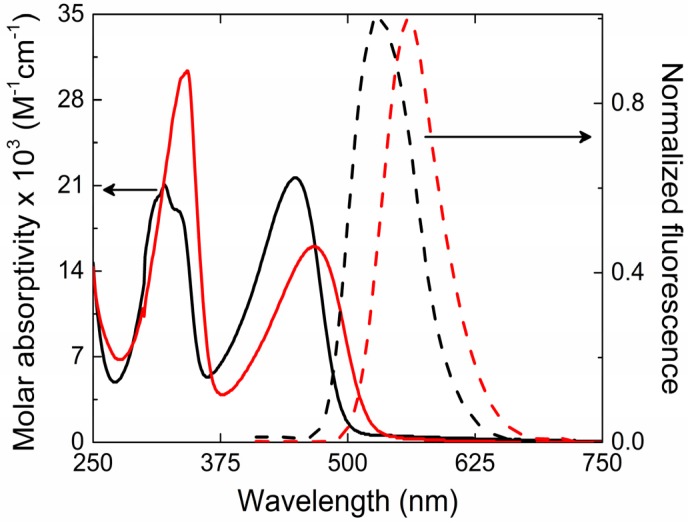
The solid and dashed lines show the linear absorption and fluorescence spectra for the PFeBT (black lines) and PFeBSe (red lines) polymers.

The ground state absorption and fluorescence spectra for PFeBT (solid lines) and PFeBSe (dashed lines) in chloroform solutions are illustrated in Figure 1. Such spectra present two absorption bands between 370–500 nm and 270–340 nm (lowest energy band—LB) for PFeBT; 390–540 nm and 270–340 nm (higher energy band—HB) for PFeBSe. According to [25], these two bands can be majorly ascribed to transitions from HOMO → LUMO + 3 and to the HOMO → LUMO (HOMO means highest occupied molecular orbital, and LUMO means lowest unoccupied molecular orbital). The last one is responsible for the charge transfer that occurs from the fluorene to the benzoselenadiazole and benzothiadiazole units. The molar absorptivity obtained for the lowest energy band was of 1.6 × 10^4^ L·mol^−1^·cm^−1^ for PFeBSe and 2.13 × 10^4^ L·mol^−1^·cm^−1^ for PFeBT polymers, respectively. This is the first indication of the stronger influence of the benzothiadiazole units on the electronic transition strength in PFeBT as compared to their analogous PFeBSe. However, it is observed that the PFeBSe presents a red-shift in the absorption (about 20 nm) with respect to PFeBT. According to Seferos et al. [26], this behavior is expected in π-conjugated molecules when heavy atom substitution occurs (from S to Se) and is related to the degree of aromaticity of the acceptor unit. The replacement of the sulfur for selenium atom results in an increase in the bond length between the nitrogen and the heavy atom, leading to a decrease in aromaticity. This decrease leads to a destabilization of HOMO and a greater stabilization of LUMO, shifting the absorption spectrum to a lower energy.

The intensity of the intramolecular charge transfer (ICT) band (and the molar absorptivity) is also affected by the change of the chalcogen atom. Since the internal charge transfer process depends on the presence of an electron-rich and electron-deficient segment, by exchanging the S atom by Se, the ability of the acceptor segment to separate and stabilize the charges decreases, as observed experimentally by the decreasing in intensity of the ICT band.

The steady state fluorescence emission of polymers presents a broadband emission with peak at *λ*_em_ = 530 nm (green) and 560 nm (yellow) for the PFeBT and PFeBSe, respectively. Such behaviors are characteristic of poly (fluorene) derivatives [27]. Vibronic structure on the fluorescence spectra were not observed for these polymers, suggesting a soft structure for these compounds. Linear and nonlinear optical parameters such as molar absorptivity, fluorescence quantum yield, fluorescence lifetimes, 2PA cross section, as well as other photophysical parameters are reported in Table 1.

**Figure 2:**
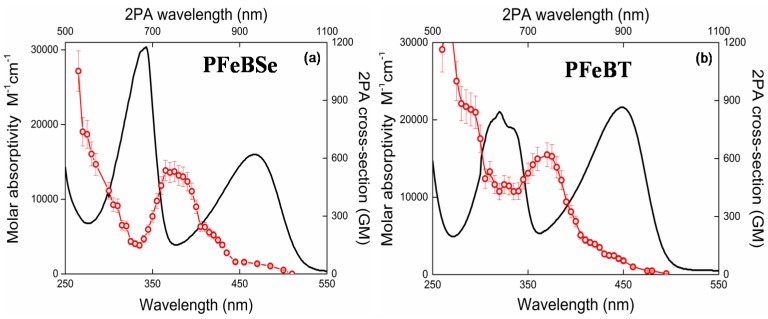
1PA (solid lines, bottom *x*-axis and left *y*-axis) and 2PA (circles, top *x*-axis and right *y*-axis) spectra for (**a**) PFeBSe and (**b**) PFeBT polymers respectively.

Figure 2 depicts the 2PA spectra for both polymers, PFeBSe (Figure 2a) and PFeBT (Figure 2b). In this figure, we also plotted the linear absorption spectrum to a better understanding of the electronic structure of the polymers. As can be seen in Figure 2, the 2PA spectra present an intense 2PA band at 360 nm (720 nm for the 2PA transition) attributed to the strongly 2PA-allowed 1Ag-like → mAg-like transition [28]. Such transition is characteristic of poly(fluorene) derivatives and can reach thousands of GM [13,28,29]. The maximum 2PA cross section obtained for this transition was 530 GM (PFeBSe) and 620 GM (PFeBT). This transition in poly(fluorene) derivatives is related to the high transition dipole moment value between the excited states, 1Bu-like and mAg-like [28]. As observed, the benzothiadiazole group causes a higher 2PA than the benzoselenadiazole group, indicating a higher π-electron delocalization for PFeBT, in agreement with the molar absorptivity data.

Below 350 nm (2 *hν* = 700 nm), we observed a considerable enhancement of the 2PA cross section because the excitation frequency approaches the one-photon-allowed lowest energy state (<550 nm), causing the intermediate state resonance enhancement effect in the 2PA transitions [30]. In this region, the 2PA cross section reaches values of 1 × 10^3^ GM.

Moreover, in Figure 2a, a monotonous decrease of the 2PA band intensity, related to the 1Ag-like → mAg-like transition, is interrupted by an increase in the 2PA cross section when the excitation photons approach the 1Ag-like → 1Bu-like (ICT) transition, indicating that there is a 2PA-allowed state in this region. Since both polymers do not present an inversion center, the 1Ag-like → 1Bu-like transition that is strongly 1PA-allowed (related to lowest energy band) may also be accessed via 2PA. This is possible because, for noncentrosymmetric molecules, the electric dipole selection rules are relaxed [31].

In addition, the 2PA band structure for this transition (1Ag-like → 1Bu-like transition) is poor because, according to the Johnsen et al. [19], the increase in conjugation length causes a structure loss in the 2PA band. Furthermore, the lowest energy 2PA band for PFeBT (97 GM) has higher cross-section values than that for PFeBSe (53 GM). This outcome suggests that there is a higher charge separation induced in PFeBT, designing a good strategy to increase the 2PA cross section in semiconductor polymers.

2PA cross section for the lowest energy transition in noncentrosymmetric molecules is directly related to the charge separation induced along the polymer chain [32,33]. Such parameter can be quantified through the difference of the permanent dipole moment between the first excited state (|1〉) and the ground state (|0〉), $\Delta {\vec{\mu }}_{01}$. Combining the solvatochromic Stokes shift measurements and the Lippert–Mataga equation we can find, $\Delta {\vec{\mu }}_{01}$, using [34]

(1) $${\left|\Delta {\vec{\mu }}_{01}^{SS}\right|}^{2}=\frac{3hc}{4\pi }\frac{\partial \upsilon }{\partial F}Vol$$

in which $\upsilon =\upsilon_{A}-\upsilon_{em}$ is the difference between the wavenumbers of the maximum absorption and fluorescence emission (in cm^−1^), $F\left(n,\xi \right)=2\left[\left(\xi -1\right)/\left(2\xi +1\right)-\left(n^{2}-1\right)/\left(2n^{2}+1\right)\right]$ is the Onsager polarity function with ξ being the dielectric constant of the solvent, and *Vol* is the volume of the effective spherical cavity occupied by the molecule inside the dielectric medium. Various solvents or mixtures of solvents (toluene, toluene/chloroform (50%/50%), chloroform, chloroform/dichloromethane (50%/50%), tetrahydrofuran (THF), and dichloromethane) with different polarities were used to investigate the charge transfer in both polymers.

**Figure 3:**
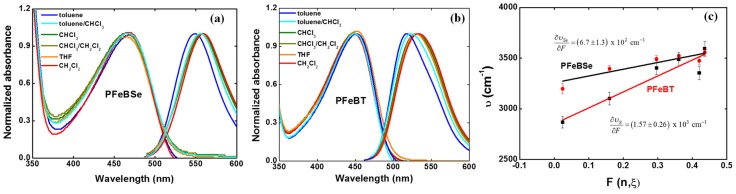
Solvatochromic Stokes shift (υ) measurements obtained as a function of the Onsager polarity function (F(n,ξ) ) for (**a**) PFeBSe and (**b**) PFeBT in a series of different solvents (toluene, toluene/chloroform (50%/50%), chloroform, chloroform/dichloromethane (50%/50%), tetrahydrofuran (THF), and dichloromethane). (**c**) Solvatochromic measurements for both polymers illustrating the increase in Stokes shift with the increase in solvent polarity.

Figure 3 depicts the solvatochromic Stokes shift measurements for (a) PFeBSe and (b) PFeBT in different solvents as well as their respective variation coefficient (∂υ/∂F, Figure 3c).

As can be seen, the lowest energy transition (1Ag-like → 1Bu-like) presents a red-shift as a function of the increase in solvent polarity, confirming the ICT character of this transition as previously mentioned. From Figure 3c, we determined $\frac{\partial \upsilon_{PFeBSe}}{\partial F}=\left(6.7\pm 1.3\right)\times 10^{2}{\;\mathrm{cm}}^{-1}$ and $\frac{\partial \upsilon_{\mathrm{PFeBT}}}{\partial F}=\left(1.57\pm 0.26\right)\times 10^{3}\;{\mathrm{cm}}^{-1}$. However, obtaining $\Delta {\vec{\mu }}_{01}$ directly from Equation (1) results in a high amount of error, which is associated with the estimation for the hydrodynamic volume of the polymer. However, the Lippert–Mataga solvatochromic approach allows us to make a comparison between the substituent groups that cause larger charge separations at the excited states along the polymer backbone. Nevertheless, in the context of the two-energy level approach for 2PA, the value of the permanent dipole moment change can be obtained from the 2PA cross section correspondent to the peak of this same transition. Therefore, we can evaluate the maximum permanent dipole moment corresponding to the ICT transition by using [35]

(2) $$\left|\Delta {\vec{\mu }}_{ICT}^{\max}\right|={\left(\frac{5}{2{\left(2\pi \right)}^{3}}\frac{N_{A}hc}{3\times 10^{3}\ln\left(10\right)}\frac{n}{L^{2}}\frac{\omega_{01}}{\varepsilon_{\max}\left(\omega_{ICT}\right)}\sigma_{ICT}^{\left(2PA-\max\right)}\left(\omega_{ICT}\right)\right)}^{1/2}$$

in which *h* is Planck’s constant, *c* is the speed of light, $\Delta {\vec{\mu }}_{ICT}^{\max}$ is the maximum difference between the permanent dipole moment corresponding to ICT transition. $L=3n^{2}/\left(2n^{2}+1\right)$ is the Onsager local field factor introduced to take into account the medium effect with *n* = 1.49 for chloroform at 20 °C. $\varepsilon_{\max}\left(\omega_{01}\right)$ is the molar absorptivity in the frequency ($\omega_{01}$) of the peak of the lowest energy 1PA band, and $N_{A}$ is the Avogadro’s number. Such value corresponds to the maximum value because we introduce the maximum values for the molar absorptivity and 2PA cross section. Proceeding in this way, we found $\Delta {\vec{\mu }}_{ICT}^{\max}=12.0D$ for PFeBSe and $\Delta {\vec{\mu }}_{ICT}^{\max}=14.3D$ for PFeBT. These results indicate clearly that there is a higher charge redistribution at the excited state when compared to the ground state for the PFeBT polymer, as previously revealed by the 2PA data.

## 3. Materials and Methods 

### 3.1. Synthesis

**Figure 4:**
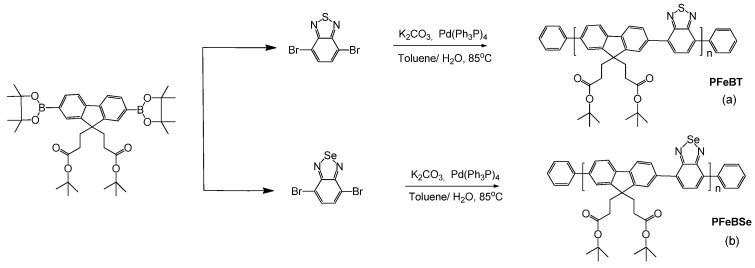
Synthesis of the donor-acceptor copolymers. (**a**) PFeBT, poly[9,9-bis(3’-(tert-butyl propanoate))fluorene-co-4,7-(2,1,3-benzothiadiazole)]. (**b**) PFeBSe, poly[9,9-bis(3’-(tert-butyl propanoate))fluorene-co-4,7-(2,1,3-benzoselenadiazole)].

The synthesis of PFeBT and PFeBSe (Figure 4) was performed according to the procedure reported in [25], which briefly consists of adding 0.31 mmol 4,7-dibromo-2,1,3-benzothiadiazole (or 4,7-dibromo-2,1,3-benzoselenadiazole), 0.31 mmol 2-(4,4,5,5-Tetramethyl-1,3,2-dioxaborolan-2-yl)-9,9bis (3-(tert-butyl propanoate)) fluorene, 8 mg of Pd(Ph3P)4, and 3.7 mmol of potassium carbonate to a 25 mL flask under an Ar atmosphere. Then, a mixture of water and toluene was added to the flask, and Ar was bubbled into the system for 20 min. The mixture was heated to 90 °C for 24 h under Ar and, after this period, 1,4-dibromobenzene and 1,4-Bis(4,4,5,5-Tetramethyl-1,3,2-dioxaborolan-2-yl) benzene were added to the reaction separately. Then, the product was precipitated in methanol, filtered, and washed with methanol and acetone. Purification was performed by Sohxlet extraction, yielding a fibrous yellow solid (PFeBT) or a fibrous orange solid (PFeBSe). PFeBT: ^1^H NMR (CDCl_3_, δ): 8.18−7.98 (br, 8H); 2.53 (br, 4H); 1.81 (m, 4H); 1.35 (s, 18H). GPC: *M*_n_ = 34,000 g·mol^−1^; PDI = 2.8. PFeBSe: ^1^H NMR (CDCl_3_, δ): 8.11−7.94 (br, 8H); 2.53 (br, 4H); 1.79 (m, 4H); 1.33 (s, 18H). GPC: *M*_n_ = 37,000 g·mol^−1^; PDI = 2.5.

### 3.2. Linear Optical Properties

In order to measure the two-photon absorption spectrum, both polymers were dissolved in chloroform with a molar concentration of approximately 1.7 × 10^−3^ M. This concentration was diluted to approximately 10^−5^ M in order to obtain linear absorption and fluorescence spectra. In addition, to calculate the 2PA cross section, we adopted the molecular weight of the monomer, i.e., 554.7 g·mol^−1^ (PFeBT) and 601.59 g·mol^−1^ (PFeSe). The UV-VIS-IR spectrum was measured by using a SHIMADZU UV-1800 (SHIMADZU, Kyoto, Japan) and a 1.0 mm optical path length quartz cuvette. Fluorescence emission was measured in a 1.0 cm optical path length quartz cuvette by using a HITACHI F7000 fluorimeter (Hitachi, Tokyo, Japan). Solvatochromic Stokes shift measurements were also performed using both equipment previously cited. For these measurements, both polymers were dissolved in four different solvents—toluene, chloroform, THF, and dichloromethane—and in a mixture of solvents—toluene/chloroform (50%/50%) and chloroform/dichloromethane (50%/50%). The concentration was kept very low (<10^−5^ M) to avoid re-absorption of the emission by the polymer, which could shift the maximum fluorescence signal to longer wavelengths.

### 3.3. Two-Photon Absorption 

The nonlinear absorption was measured by means of a femtosecond wavelength-tunable open aperture Z-scan technique [36]. Our Z-Scan setup used a Ti/sapphire laser amplifier (Clark-MXR, Inc., Michigan, USA) with a 1 kHz repetition rate and a pulse width of about 150 fs. This laser pumps an optical parametric amplifier (TOPAS) (Light Conversion ,Vilnius, Lithuania) that delivers pulses covering the wavelength ranged from 460 nm up to 2000 nm. Specifically on this work, TOPAS was wavelength-tuned between 500 nm up 1100 nm. At this range of wavelength, the pulse width was evaluated to approximately 120 fs, obtained by measuring optical kerr gate effect on a 1 mm fused silica. Once the wavelength was set, it passes through a 50 μm diamond spatial filter located at the focal region of a 1 m telescope. This step is important to filter spatially the beam and achieve a better Gaussian spatial profile. After that, the laser beam is aligned to the Z-Scan line, in which a beam splitter is used to reflect 4% of the laser irradiance to a 1 cm^2^ silicon detector (reference). The reference detector is used to normalize the measurement for the laser fluctuation. In order to control the laser average power and fix the polarization of the beam, two calcite polarizers are used. A convergent lens with *f* = 15 cm is employed to generate the nonlinear effect due to the high intensity at the focal region. A second lens is used to focus the beam at the second photodetector (1 cm^2^), which is responsible for monitoring the nonlinear absorption induced on the sample. Both detectors are coupled to independent locking amplifiers, in which the electrical signal is amplified and integrated. Afterwards, the signal from the second detector is normalized to the reference detector in order to remove the laser fluctuation from the nonlinear measurements. The Z-scan measurements were carried out with averaged power ranging from 50 to 100 μW and a beam waist size at the focus varying from 14 to 19 μm.

In order to verify if photodecomposition could be generated because of the high intensity of the laser during the Z-Scan measurement, the linear absorption spectrum was measured after measurements and compared with a reference spectrum.

**Figure 5:**
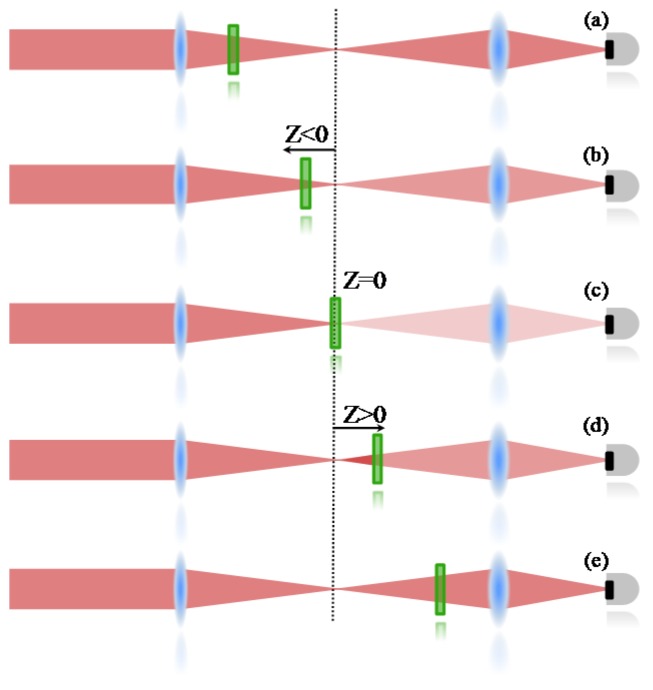
Schematic representation of the transmittance changes as the sample is translated through the focal plane of a focused Gaussian beam. In (a) and (e), sample is far from the focus and there is no nonlinear absorption. For (b) and (d), the sample is close to the focal plane, at this region the intensity may induce weak nonlinear absorption. At *Z* = 0 (c), the nonlinear absorption effect is the maximum achieved.

An open aperture Z-Scan was used to measure the two-photon absorption, whose details can be found elsewhere [36]. Therefore, only a summarized description will be given here. In the Z-scan technique, it is important to know that nonlinear effects induced on the sample change the transmittance of the beam, which is monitored by a detector at the far field. Therefore, the 2PA cross section is determined when the sample is translated through the focal plane of a focused Gaussian beam; if the sample is far from the focal point (Figure 5a), the intensity of the laser is not enough to promote a transition mediated by the absorption of two photons. Consequently, the transmittance is not changed at the detector. When the sample is close to the focal region (Figure 5b), the intensity increases and some 2PA starts to take place, decreasing the number of photons that achieve the detector (decreasing the transmittance). At the focal point (Figure 5c), the intensity is the highest and the nonlinear absorption reaches its maximum, decreasing the transmittance to the lowest level. After the sample passes to the focal point (Figure 5d,e), the effect induced will be similar to the ones observed before the focal point, giving a symmetrical transmittance signature with respect to the focal region (*Z* = 0).

In the Z-scan experiment, the light field creates an intensity-dependent absorption, α = α_0_ + β*I*, in which α_0_ is the linear absorption coefficient, which is zero in most cases, *I* is the laser beam intensity, and β is the 2PA coefficient (nonlinear absorption coefficient). β is obtained by fitting the Z-Scan measurement. 

Far from one-photon resonances, the power transmitted through the sample due to a 2PA process, for each wavelength, is integrated over time (assuming a pulse with a Gaussian temporal profile) to give the normalized energy transmittance:

(3) $$T(z)=\frac{1}{\sqrt{\pi }q_{o}\left(z,0\right)}\int_{-\infty }^{\infty }\ln\left[1+q_{o}(z,0)e^{-\tau^{2}}\right]d\tau$$

where

(4) $$q_{o}=\beta I_{o}L{\left(1+\left(z^{2}/z_{o}^{2}\right)\right)}^{-1}$$

in which *L* is the sample thickness, *z*_0_ is the Rayleigh length, *z* is the sample position, and *I*_0_ is the laser intensity at the focus. The nonlinear coefficient β is obtained by fitting the Z-scan data with Equation (1). The 2PA cross section, $\sigma_{2PA}$, is determined from $\sigma_{2PA}=\hbar \omega \beta /N$, in which ℏω is the excitation photon energy, and *N* is the number of molecules per cm^3^. Usually, the 2PA cross section is expressed in units of Göppert–Mayer (GM) (1 GM = 1 × 10^−50^ cm^4^·s·photon^−1^). 

### 3.4. Time-Resolved Fluorescence

Fluorescence decay time was measured by exciting both polymers at approximately 390 nm. The wavelength of excitation was generated by using a BBO thin crystal, in which a 780 nm laser, delivered by CPA Ti/sapphire laser with a 1 kHz repetition rate and a pulse width of about 150 fs, was doubled in frequency. The fluorescence signal was collected by an optical fiber with a 1 mm core diameter and delivered at a Silicon fast photodetector (rise time: ~0.7 ns). Two optical filters were used: one to remove the 780 nm after the BBO crystal and the other to remove the 390 nm from the fluorescence signal. Both fluorescence decay times were analyzed by the de-convoluting method, in which the response time of the detector was used.

## 4. Conclusions

Here, we have studied femtosecond 2PA spectrum for two push–pull poly(fluorene) derivatives containing benzoselenadiazole and benzothiadiazole units. Both push–pull polymers present considerable 2PA cross sections (up to 10^3^ GM), with one higher energy 2PA band associated with the strongly 2PA-allowed transition (1Ag-like → mAg-like) and another one with the lowest energy related to an ICT transition. The later one is also observed along to the one-photon absorption spectrum due to the relaxation of the electric dipole selection rules in noncentrosymmetric materials. Besides that, our results pointed out that the PFeBT polymer presents a higher 2PA cross section as compared to the PFeBSe, along the entire nonlinear spectrum. These outcomes are related to the larger charge redistribution in the PFeBT polymer as compared to their analogous PFeBSe, occasioned by the increase of the electron-withdrawing group strength (from Se to S), corroborated through the Stokes shift measurements. Our results show that the push–pull copolymers are a good molecular strategy to develop remarkable materials for 2PA.

## Figures and Tables

**Table 1:** Photophysical data of Chromophores 1–3 (in chloroform).

Polymers	PFeBSe	PFeBT
$\lambda_{1^{0}peak}^{1PA-abs}$ (nm)	467	449
$\lambda_{2^{0}peak}^{1PA-abs}$ (nm)	342	325
$\varepsilon_{LB}^{\max}$ (M^−1^·cm^−1^)	1.6 × 10^4^	2.13 × 10^4^
$\varepsilon_{HB}^{\max}$ (M^−1^·cm^−1^)	3.02 × 10^4^	2.02 × 10^4^
$\Delta {\vec{\mu }}_{ICT}$ (D)	12.0	14.3
$\sigma_{LB}^{\left(2PA\right)}$ (GM)	53	97
$\sigma_{HB}^{\left(2PA\right)}$ (GM)	530	620
$\lambda_{\max}^{em}$ (nm)	558	530
Φ_f_ (%)	1.6	3.9
τ (ns)	1.2 ± 0.1	2.1± 0.1

$\lambda_{1^{0}peak}^{1PA-abs}$ and $\lambda_{2^{0}peak}^{1PA-abs}$ are the wavelengths correspondents to the first and second peak present in the linear absorption spectra; $\sigma_{LB}^{\left(2PA\right)}$ and $\sigma_{HB}^{\left(2PA\right)}$ are the 2PA cross section related to the first (low energy) and second (high energy) 2PA band observed along the nonlinear optical spectra.

## References

[1] Patil A.O., Heeger A.J., Wudl F. (1988). Optical-properties of conducting polymers. Chem. Rev..

[2] Sun H.B., Mizeikis V., Xu Y., Juodkazis S., Ye J.Y., Matsuo S., Misawa H. (2001). Microcavities in polymeric photonic crystals. Appl. Phys. Lett..

[3] Ma H., Jen A.K.Y., Dalton L.R. (2002). Polymer-based optical waveguides: Materials, processing, and devices. Adv. Mater..

[4] Burroughes J.H., Bradley D.D.C., Brown A.R., Marks R.N., Mackay K., Friend R.H., Burn P.L., Holmes A.B. (1990). Light-emitting-diodes based on conjugated polymers. Nature.

[5] Friend R.H., Gymer R.W., Holmes A.B., Burroughes J.H., Marks R.N., Taliani C., Bradley D.D.C., dos Santos D.A., Bredas J.L., Logdlund M. (1999). Electroluminescence in conjugated polymers. Nature.

[6] Decher G. (1997). Fuzzy nanoassemblies: Toward layered polymeric multicomposites. Science.

[7] Maruo S., Nakamura O., Kawata S. (1997). Three-dimensional microfabrication with two-photon-absorbed photopolymerization. Opt. Lett..

[8] Moughames J., Jradi S., Chan T.M., Akil S., Battie Y., Naciri A.E., Herro Z., Guenneau S., Enoch S., Joly L. (2016). Wavelength-scale light concentrator made by direct 3D laser writing of polymer metamaterials. Sci. Rep..

[9] Henrique F.R., Mendonca C.R. (2016). Local excitation and collection in polymeric fluorescent microstructures. Opt. Mater..

[10] Estevam-Alves R., Ferreira P.H.D., Almeida G.F.B., Sousa W.S., Mendonca C.R. (2014). Microfabrication of electroluminescent polymer for devices construction. Appl. Surf. Sci..

[11] Shen Y., Swiatkiewicz J., Prasad P.N., Vaia R.A. (2001). Hybrid near-field optical memory and photofabrication in dye-doped polymer film. Opt. Commun..

[12] Orlic S., Ulm S., Eichler H.J. (2001). 3D bit-oriented optical storage in photopolymers. J. Opt. A-Pure Appl. Opt..

[13] Correa D.S., de Boni L., Nowacki B., Grova I., Fontes B.D., Rodrigues P.C., Tozoni J.R., Akcelrud L., Mendonca C.R. (2012). Two-photon excitation and optical limiting in polyfluorene derivatives. J. Polym. Sci. Part B-Polym. Phys..

[14] Poornesh P., Hegde P.K., Umesh G., Manjunatha M.G., Manjunatha K.B., Adhikari A.V. (2010). Nonlinear optical and optical power limiting studies on a new thiophene-based conjugated polymer in solution and solid PMMA matrix. Opt. Laser Technol..

[15] Belfield K.D., Ren X.B., van Stryland E.W., Hagan D.J., Dubikovsky V., Miesak E.J. (2000). Near-IR two-photon photoinitiated polymerization using a fluorone/amine initiating system. J. Am. Chem. Soc..

[16] Tsiminis G., Ruseckas A., Samuel I.D.W., Turnbull G.A. (2009). A two-photon pumped polyfluorene laser. Appl. Phys. Lett..

[17] Pawlicki M., Collins H.A., Denning R.G., Anderson H.L. (2009). Two-Photon Absorption and the Design of Two-Photon Dyes. Angew. Chem. Int. Ed..

[18] Kuzyk M.G. (2003). Fundamental limits on two-photon absorption cross sections. J. Chem. Phys..

[19] Johnsen M., Paterson M.J., Arnbjerg J., Christiansen O., Nielsen C.B., Jorgensen M., Ogilby P.R. (2008). Effects of conjugation length and resonance enhancement on two-photon absorption in phenylene-vinylene oligomers. Phys. Chem. Chem. Phys..

[20] Vivas M.G., Silva D.L., Malinge J., Boujtita M., Zalesny R., Bartkowiak W., Agren H., Canuto S., de Boni L., Ishow E. (2014). Molecular Structure—Optical Property Relationships for a Series of Non-Centrosymmetric Two-photon Absorbing Push–pull Triarylamine Molecules. Sci. Rep..

[21] Orr B.J., Ward J.F. (1971). Perturbation Theory of Non-Linear Optical Polarization of an Isolated System. Mol. Phys..

[22] Lee S., Thomas K.R.J., Thayumanavan S., Bardeen C.J. (2005). Dependence of the two-photon absorption cross section on the conjugation of the phenylacetylene linker in dipolar donor-bridge-acceptor chromophores. J. Phys. Chem. A.

[23] Patra A., Bendikov M. (2010). Polyselenophenes. J. Mater. Chem..

[24] Tsoi W.C., James D.T., Domingo E.B., Kim J.S., Al-Hashimi M., Murphy C.E., Stingelin N., Heeney M. (2012). Effects of a Heavy Atom on Molecular Order and Morphology in Conjugated Polymer: Fullerene Photovoltaic Blend Thin Films and Devices. ACS Nano.

[25] Rodrigues P.C., Berlim L.S., Azevedo D., Saavedra N.C., Prasad P.N., Schreiner W.H., Atvars T.D.Z., Akcelrud L. (2012). Electronic Structure and Optical Properties of an Alternated Fluorene-Benzothiadiazole Copolymer: Interplay between Experimental and Theoretical Data. J. Phys. Chem. A.

[26] Gibson G.L., McCormick T.M., Seferos D.S. (2012). Atomistic Band Gap Engineering in Donor-Acceptor Polymers. J. Am. Chem. Soc..

[27] Cornil J., Gueli I., Dkhissi A., Sancho-Garcia J.C., Hennebicq E., Calbert J.P., Lemaur V., Beljonne D., Bredas J.L. (2003). Electronic and optical properties of polyfluorene and fluorene-based copolymers: A quantum-chemical characterization. J. Chem. Phys..

[28] Najechalski P., Morel Y., Stephan O., Baldeck P.L. (2001). Two-photon absorption spectrum of poly(fluorene). Chem. Phys. Lett..

[29] De Boni L., Fonseca R.D., Cardoso K.R.A., Grova I., Akcelrud L., Correa D.S., Mendonca C.R. (2014). Characterization of Two- and Three- Photon Absorption of Polyfluorene Derivatives. J. Polym. Sci. Part B-Polym. Phys..

[30] Hales J.M., Hagan D.J., van Stryland E.W., Schafer K.J., Morales A.R., Belfield K.D., Pacher P., Kwon O., Zojer E., Bredas J.L. (2004). Resonant enhancement of two-photon absorption in substituted fluorene molecules. J. Chem. Phys..

[31] Daoud M., Kibler M. (1995). 2-Photon spectroscopy between states of opposite parities. Phys. Rev. B.

[32] Badaeva E.A., Timofeeva T.V., Masunov A.M., Tretiak S. (2005). Role of donor-acceptor strengths and separation on the two-photon absorption response of cytotoxic dyes: A TD-DFT study. J. Phys. Chem. A.

[33] Drobizhev M., Makarov N.S., Rebane A., de la Torre G., Torres T. (2008). Strong two-photon absorption in push–pull phthalocyanines: Role of resonance enhancement and permanent dipole moment change upon excitation. J. Phys. Chem. C.

[34] Suppan P. (1990). Solvatochromic shifts—The influence of the medium on the energy of electronic states. J. Photochem. Photobiol. A..

[35] Vivas M.G., de Boni L., Cooper T.M., Mendonca C.R. (2014). Understanding the Two-Photon Absorption Spectrum of PE2 Platinum Acetylide Complex. J. Phys. Chem. A.

[36] Sheik-Bahae M., Said A.A., Wei T.-H., Hagan D.J., Stryland E.W.V. (1990). Sensitive Measurement of Optical Nonlinearities Using a Single Beam. IEEE J. Quantum Electron..

